# Parental phubbing and academic burnout in adolescents: the role of social anxiety and self-control

**DOI:** 10.3389/fpsyg.2023.1157209

**Published:** 2023-05-18

**Authors:** Yuqian Jiang, Lu Lin, Ronghua Hu

**Affiliations:** School of Educational Science, Anhui Normal University, Wuhu, China

**Keywords:** parental phubbing, academic burnout, social anxiety, self-control, adolescents

## Abstract

Based on the limited resource model of self-control, we construct a chain mediation model to examine the relationship between parental phubbing and adolescents’ academic burnout, and whether social anxiety and self-control play a mediating role in it. We used 4 questionnaires to investigate parental phubbing, social anxiety, self-control, and adolescents’ academic burnout among 828 high school students in Wuhu and Huangshan City, Anhui Province, China. The findings indicated that: (1) parental phubbing, social anxiety, and self-control all significantly predict adolescents’ academic burnout directly and (2) parental phubbing could indirectly influence adolescents’ academic burnout through three pathways: the separate mediating effect of social anxiety and self-control, and the chain mediating effect on both. The results of this study help parents understand how their phubbing actions affect adolescents’ academic burnout and the mechanism of action.

## 1. Introduction

With the fast expansion of internet communication devices, these devices such as cell phones and iPads have become more and more feature-rich, and all aspects of life are filled with them. The emergence of functions such as online shopping, takeout, and even online grocery shopping has made people today increasingly inseparable from their devices. And the emergence of the WeChat work group has also allowed people to respond to work messages timely even from home. However, researchers have also found that the overuse of mobile communication devices has a negative impact on users’ daily lives (Lee et al., 2014; Lin et al., 2016). Nowadays, it is common for parents to use cell phones in the family, but it is not well understood whether such parental behavior has a negative impact on adolescents, especially regarding adolescents’ academic burnout. Most previous studies on the effects of cell phone use on academic burnout have focused on the behaviors of adolescents themselves (Gong et al., 2021). However, from the perspective of parents to explore how their phubbing actions affect adolescents’ academic burnout and how it works has not been emphasized. Therefore, the present study aimed to examine the effects and potential mechanisms of parental cell phone use (parental phubbing) on adolescents’ academic burnout.

In recent years, excessive cell phone use has become a widespread societal issue, giving rise to the term “phubbing,” which refers to the phenomenon of individuals who are too preoccupied with their mobile phone or iPad to ignore others (Chotpitayasunondh and Douglas, 2016). Phubbing can occur on any social occasion, such as between coworkers, friends, couples, and parents and children, but regardless of the social occasion, it will decrease the quality of social interactions perceived by the phubbers (Chotpitayasunondh and Douglas, 2016; Chotpitayasunondh and Douglas, 2018; Beukeboom and Pollmann, 2021). Studies have found that simply recalling a situation in which a partner is playing with a phone in front of them can cause individuals to feel excluded (Hales et al., 2018). Parents may be interrupted by external messages such as phone calls and text messages during parent–child interactions, or neglect their children due to the attraction of the phone’s content. This kind of “phubbing” that occurs during parent–child interaction is known as “parental phubbing” (Wang and Qiao, 2022).

It is undeniable that parents are the main factor influencing adolescent development. Based on the Ecological System Theory, the most significant microsystem influencing adolescent development is the family, and as parents are the primary members of the family, their behavior has a significant influence on adolescent development (Bronfenbrenner, 1979; Bronfenbrenner and Ceci, 1994). Studies have shown that parents playing with their cell phones in front of their children will affect the quality of communication and interaction in the family (Mcdaniel and Coyne, 2016; Mcdaniel and Radesky, 2018a). This in turn affects the parent–child relationship (Kildare and Middlemiss, 2017). Sharaievska and Stodolska (2017) found that parents’ use of cell phones during family leisure time leads to resentment from other family members who complain that they only love their phones and do not love them. Children may perceive electronic devices in their parents’ hands as a threat to compete with them for their parents’ favor, which leads to feelings of jealousy (Clayton et al., 2013). This also leads to an increased likelihood of family conflict. According to attachment theory, when parents are too addicted to their devices during parent–child interactions, the development of secure attachments between children and their parents may be affected by unmet needs for closeness (Radesky and Christakis, 2016; Sabha, 2022). Mcdaniel and Radesky (2018b) suggests that we should be vigilant about parents’ use of cell phones in daily life with their children because parents may suddenly lose interest in parenting their children due to being preoccupied with their phones, a pattern he calls “distracted parenting.”

Parental phubbing is perceived by children as a type of carelessness and refusal (Roberts and David, 2016; David and Roberts, 2017), while Pinquart (2016, 2017) found that neglectful parenting and a lack of parental responsiveness were linked to poorer academic performance in children. Investigating whether parental phubbing affects adolescents’ academic burnout is therefore both theoretically interesting and practically useful.

### 1.1. Parental phubbing and adolescents’ academic burnout

Academic burnout is a chronic psychological disorder in which students experience physical and mental exhaustion and poor academic achievement (Salmela-Aro et al., 2009; Wu et al., 2010; Zhang et al., 2021). Academic burnout can lead to many school adjustments problems, like decreased academic performance, truancy, internet addiction, and depression (Wang et al., 2015; Tang et al., 2021). In severe cases, it can lead to physical illnesses such as hypertension and atherosclerosis (May et al., 2014).

Family environment affects adolescents’ levels of academic burnout (Luo et al., 2020). Parents are the most significant social support system for children (Kochanska and Aksan, 2004; Pinquart, 2016; Pinquart, 2017). When parents play on their phones in front of children and ignore them, children experience decreased parental support and warmth and more severe feelings of rejection, neglect, and parental rejection (Roberts and David, 2016; Stockdale et al., 2018). Previous research has found that adolescents who are neglected and rejected are more likely to experience academic burnout (Luo et al., 2016). In addition, parental phubbing can reduce parent–child relationships (Kildare and Middlemiss, 2017; Niu et al., 2020), while negative parent–child relationships have been found to be an important predictor of adolescents’ academic burnout (Pinquart, 2016). And He et al. (2022) found that parental phubbing significantly predicted secondary school students’ academic burnout as well. Therefore, we proposed the hypothesis that parental phubbing is positively associated with adolescents’ academic burnout (H1).

### 1.2. The mediating role of social anxiety

Based on Ecological System Theory (Bronfenbrenner, 1979; Bronfenbrenner and Ceci, 1994), in addition to family factors, peers are an important microsystem that influences children’s development. After adolescence, the influence of peers on individuals gradually increases as adolescents become more autonomous and independent, and becomes an important environmental factor affecting the development of adolescents (Brown and Bakken, 2011; Jewell and Brown, 2014). Previous research has found that poor peer relationships would result in lower academic performance in adolescents (Woodward and Fergusson, 2000), and social anxiety is an inherent manifestation of poor peer relationships (La Greca and Lopez, 1998).

Social anxiety is one of the most prevalent anxiety symptoms in adolescents and is characterized by a strong and irrational feeling of embarrassment in social situations (Morrison and Heimberg, 2013; Alkozei et al., 2014). Individuals with social anxiety fear negative judgments and always try to avoid being around others (Watson and Friend, 1969). The family environment is one of the major contributors to social anxiety, and as parents are the most important family members, their behavior will have a large impact on the level of their children’s social anxiety (Gómez-Ortiz et al., 2019). Communication and parental responses are crucial to children and adolescents’ development in the parent–child relationship (Kochanska and Aksan, 2004; Pinquart, 2016; Pinquart, 2017). When parents are distracted by their cell phones at home, children will experience less parental warmth and support (Stockdale et al., 2018), reduce the quality of communication (Chotpitayasunondh and Douglas, 2016, 2018), and affect the parent–child relationships (Kildare and Middlemiss, 2017). Adolescents’ development of positive interpersonal interactions and family closeness is significantly influenced by parent–child communication. Reduced parent–child communication and relationships will prevent children from developing the attitudes and social skills necessary to get along with others in a healthy way, as well as create unfavorable expectations for interpersonal interactions, which will in turn cause social anxiety in children. In addition, it has also been found that parental phubbing directly predicts adolescents’ social anxiety levels (Zhang et al., 2022).

Adolescents with social anxiety shy away from speaking up in class discussions and talking with classmates outside of class, and are afraid to ask teachers and peers for help when they are struggling academically, consequently leading to learning problems. Bernstein et al. (2008) found high levels of social anxiety are linked to poor social skills, attention disorders, and academic challenges in school. Russell and Topham (2012) also reviled social anxiety could affect college students’ learning and well-being. More extensive research has revealed that social anxiety raises the chance of exam failure and early school exit, which results in failure to graduate (Stein and Kean, 2000). More recently, researchers have also found that social anxiety could negatively predict students’ academic engagement (Mou et al., 2022). Academic engagement may be seen as academic burnout’s antithesis since researchers discovered a strong negative association between the two (Alarcon et al., 2011). With the above analysis, we expect that parental phubbing could increase adolescents’ academic burnout by raising their social anxiety (H2).

### 1.3. The mediating role of self-control

The preceding discussion explained the relationship between parental phubbing, social anxiety, and adolescent academic burnout, but there exists another key factor that could affect adolescents’ learning. Researchers have found that self-control is a protective factor for academic burnout (Seibert et al., 2016; Love et al., 2020). Actually, self-control is a protective factor for many problem behaviors and mental health issues, those with high self-control have relatively low addictive behaviors (Baumeister and Vonasch, 2015), criminal behavior (Flexon et al., 2016a), as well as anxiety and depression (Oliva et al., 2019). However, in this study, we did not want to discuss the protective effect of self-control on academic burnout. The purpose of this study was to find out whether this protective factor for academic burnout could be influenced by other variables. In other words, we wanted to know whether parental phubbing and social anxiety would affect adolescents’ academic burnout by reducing self-control.

Self-control is the capacity that individuals to suppress or curb their desires and impulses and regulate their inherent ways of thinking or behavioral habits to conform to social norms, such as resisting temptation, delaying gratification, and resisting impulses (Muraven et al., 1998; Tang and Guo, 2008). In adolescent development, there are many problem behaviors such as substance abuse, violence, smoking, cell phone addiction, and truancy, which can be theoretically attributed to the failure of self-control (Flexon et al., 2016b; Mei et al., 2016). The Limited Resource Model of Self-control (Muraven et al., 1998; Baumeister et al., 2007) suggests that, like physical strength, the capacity for self-control seems to be a finite resource that will be depleted throughout the self-regulation process, and that a lack of control resources will result in control failure.

The act of learning requires a large number of control resources, and people must exercise more self-control during learning in order to suppress the interference of irrelevant information and maintain their attention on the learning task (Tangney et al., 2004; He et al., 2017). That is, individuals with poorer self-control experienced greater levels of academic burnout.

Self-control can be influenced by the environment (Beaver et al., 2009), for external environmental factors would deplete an individual’s self-control resources, which results in a decrease in self-control. Previous studies have found that parenting styles (Li et al., 2019), school environments (Turner et al., 2005), and peer relationships (Meldrum et al., 2012) can all affect adolescent self-control. Warmer parenting, more emotional support, close parent–child relationships, and secure parent–child attachments are correlated with greater self-control in children (Botchkovar et al., 2015; Li et al., 2019). In contrast, low warmth parenting, more child neglect, and less parent–child interaction are correlated with lower self-control (Davis et al., 2017; Li et al., 2019). While parental phubbing is perceived by children as a type of carelessness and refusal (Roberts and David, 2016), which lead them to experience lower warmth and support (Stockdale et al., 2018). Therefore, it is reasonable to expect that parental phubbing could increase adolescents’ academic burnout by decreasing their self-control (H3).

### 1.4. The sequential mediation effect of social anxiety and self-control

Except for the external environment (parental phubbing in this study), the individual’s negative emotional state (social anxiety in this study) can also deplete the individual’s self-control resources (Blackhart et al., 2015). Social anxiety is a negative emotion that causes people to feel fear and tension in social situations (Alkozei et al., 2014). Therefore, individuals need to expend resources of self-control to overcome these bad feelings. Kashdan et al. (2011) found individuals with high social anxiety exert more effort to self-regulate during social activities than those with low. Blackhart et al. (2015) found for socially anxious people, simply communicating with others would deplete self-control resources, leading to reducing the effect of subsequent self-regulation. According to the Limited Resource Model of Self-control mentioned above, as a limited resource, self-control will be consumed similarly to muscle power (Muraven et al., 1998; Muraven and Baumeister, 2000; Baumeister et al., 2007). Meanwhile, the act of learning itself requires significant consumption of control resources. That is, when the limited resources for self-control are occupied by environmental factors and negative emotional states, the remaining resources are insufficient to cope with the needs of learning, which leads to learning problems. Above all, we hypothesized that social anxiety and self-control play a chain mediating role between parental phubbing and adolescents’ academic burnout (H4).

**Figure 1:**
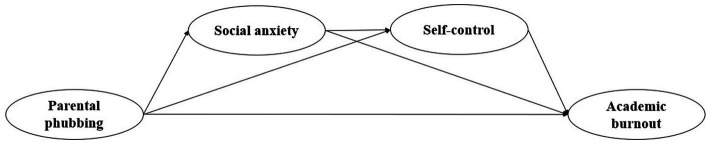
The hypothetic mediation model.

In conclusion, previous studies have found that parental phubbing will lead to academic burnout in adolescents (He et al., 2022), but the mechanisms of action need to be further explored. Investigating the mechanisms of parental phubbing on academic burnout in adolescents can help parents understand possible ways to reduce adolescent learning problems. Researchers have found that inadequate self-control resources are an important cause of academic burnout (He et al., 2017; Love et al., 2020). While both the external environment and negative emotional state would deplete individuals’ self-control resources and lead to reduced self-control (Beaver et al., 2009; Blackhart et al., 2015). Consequently, based on the Limited Resource Model of Self-control, we proposed a chain mediation model (Figure 1) in an attempt to explore whether parental phubbing and social anxiety would affect adolescents’ academic burnout by decreasing their self-control.

## 2. Research methodology

### 2.1. Procedure and subjects

We used random cluster sampling to recruit adolescents from three full-time secondary schools in Anhui province, China. With the consent of teachers and guardians, students filled out the questionnaire during their weekly class meetings. The participants were informed of the anonymity, voluntary, and confidential nature of the questionnaire.

1,032 adolescents were recruited to participate in this study. Invalid questionnaires that failed polygraph questions and consistent responses were excluded. Finally, 828 valid questionnaires met the last criteria, with an effective rate of 80.1%. Among them, 376 (45.4%) were male, and 452 (54.6%) were female; 333 (40.2%) were grade 10, 295 (35.6%) were grade 11, and 200 (24.2%) were grade 12. Their mean age is 16.04 (SD = 0.95), ranging from 15 to 18. There were 315 (38.0%) participants who were only children and 523 (62.0%) participants who were not only children. 429 (51.8%) participants reported being urban residents and 399 (48.2%) reported being rural residents. The gender distribution of participants and their grades did not significantly correlate, *χ*^2^_(1)_ = 3.89, *p* = 0.143.

### 2.2. Instruments

#### 2.2.1. Parental phubbing scale

Using the Chines version of Parental phubbing Scale from Roberts and David (2016), as amended by Ding et al. (2020). It was a single-dimensional scale and consists of 9 items (e.g., my parents check their phones when I am talking to them), each of which was assessed on a Likert scale from 1 to 5 (1 being “never do this” and 5 being “always do this”). A higher total score means more severe parental phubbing. In this investigation, the scale showed great internal consistency reliability (Cronbach’s *α* 0.81).

#### 2.2.2. The adolescents’ academic burnout scale

Using the Adolescents’ Academic Burnout Scale developed by Wu et al. (2010) to masseur adolescents’ academic burnout. This version was modified to fit elementary to high school students. The scale had 16 items (e.g., I feel extremely tired after a day of studying) and was divided into three subclasses. A 5 point Likert scale ranging from completely disagree to completely agree was employed. The internal consistency reliability (Cronbach’s *α*) in this research was 0.85 for the total scale, and for each of the three subscales, it was 0.78, 0.85, and 0.84.

#### 2.2.3. Adolescent social anxiety scale

To measure adolescents’ social anxiety, we utilized the Chinese translation of the Adolescent Social Anxiety Scale, which was amended by Zhu (2008) from La Greca and Lopez (1998). Instead of the original 18 items, the Chinese version has been reduced to 13 items, such as “I am reluctant to invite people to do things with me because I am afraid of rejection.” The questionnaire was divided into 3 dimensions and a Likert scale from 1–5 (not at all to exactly) was used to assess. The internal consistency reliability (Cronbach’s *α*) of the total scale was 0.93 and for each of the three subscales, it was 0.92, 0.84, and 0.83.

#### 2.2.4. The self-control scale

We used the Chinese version Self-control Scale amended by Tang and Guo (2008), which was created by Tangney et al. (2004). The original version had 36 items, and the Chinese version was revised with 19 items, such as “I can work effectively for a long-term goal,” divided into 5 dimensions. The scale is rated on a Likert scale from 1 (completely inconsistent) to 5 (completely consistent). Internal consistency reliability (Cronbach’s *α*) for the overall scale was 0.89, and was 0.85, 0.66, 0.72, 0.61, and 0.72 for the 5 subscales in the current study.

### 2.3. Data analysis

Data entry, correlation analyses, and descriptive statistics were carried out using SPSS 26.0 and mediated effects analysis was performed using Mplus 8.1. We first screened the collected data and performed descriptive statistics and correlation analysis between variables after eliminating invalid data. Based on the correlation analysis, we controlled for the effects of gender and grade level and established a direct pathway of parental phubbing to adolescents’ academic burnout. To test the mediating effect, we then incorporated social anxiety and self-control in the mediation model and constructed a pathway of influence from social anxiety to self-control to generate a chain mediation model. Bootstrap testing was applied to estimate confidence intervals and test for indirect effects. And the chi-square value, RMSEA, CFI, TLI, and SRMR was used to evaluate the model fit index.

### 2.4. Common method bias test

This study used only questionnaires for administration, which may have created common method bias (CMB) due to the same data collection method. We emphasized the anonymity and confidentiality of the questionnaires to the participants before they filled out the questionnaires, and used methods such as positive and negative scoring in the questionnaires to preliminary control this bias. Further, two statistical methods were used to test for CMB. (1) Harman’s single-factor test. 20 factors were extracted with eigenvalues bigger than 1, and the first one was able to account for 16.46% of the total variation, indicating that the CMB was not serious in this study for the normal criterion of 40% was not exceeded. (2) Using confirmatory factor analysis (Harris and Mossholder, 1996) to test for CMB. We created a single-factor model and used all the items of the latent variables as new observations for this model. The findings revealed a poor fit for the single-factor model: *χ*^2^/d*f* = 9.77, RMSEA = 0.103, CFI = 0.39, TLI = 0.37, and SRMR = 0.117, suggesting that the CMB was not serious in this study.

## 3. Results

### 3.1. Correlation analysis and descriptive statistics

Table 1 displays the means, standard deviations, and coefficient of correlation for each variable. Parental phubbing, social anxiety, and academic burnout were positively associated in pairs, while self-control was negatively associated with them.

**Table 1:** Means, standard deviations, and correlations of each variable (*N* = 828).

	*M*	SD	1	2	3	4
1 Parental phubbing	2.86	0.73	1			
2 Social anxiety	2.87	0.86	0.19***	1		
3 Self-control	3.06	0.60	−0.21***	−0.33***	1	
4 Academic burnout	2.88	0.56	0.22***	0.43***	−0.56***	1

**p < 0.01, ***p < 0.001.

### 3.2. Mediation effect test

Since social anxiety, self-control, and academic burnout all contain multiple dimensions, it is necessary to pack these three scales for controlling the impacts of random errors (Wu and Wen, 2011). The specific method is to take the mean value of each subscale as the new observation index of each latent variable. For example, the burnout scale contains 3 dimensions: low achievement, academic alienation, and physical and mental exhaustion. The mean values of each 3 dimensions are used as the new observables of academic burnout.

The impact of gender and grade was controlled for getting a more accurate effect. We first tested the direct path of parental phubbing on adolescents’ academic burnout. The results revealed a good model fit: *χ*^2^/d*f* = 3.70, RMSEA = 0.057, CFI = 0.93, TLI = 0.91, SRMR = 0.039, and a significant positive impact of parental phubbing on academic burnout (*β* = 0.28, *t* = 6.23, *p* < 0.001).

**Figure 2:**
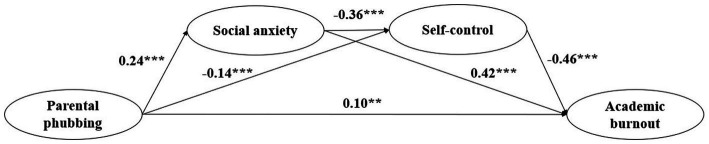
The chain mediating model of social anxiety and self-control in the relationship between parental phubbing and adolescents’ academic burnout.

Secondly, to test the chain mediation effect of social anxiety and self-control, a chain mediation model was developed. The model fit was acceptable: *χ*^2^/d*f* = 4.29, RMSEA = 0.063, CFI = 0.89, TLI = 0.87, and SRMR = 0.053. The path analysis indicated that the direct effect remained significant (*β* = 0.10, *t* = 2.50, *p* = 0.012), and parental phubbing significantly predicted social anxiety positively (*β* = 0.24, *t* = 6.10, *p* < 0.001) and self-control negatively (*β* = −0.14, *t* = −6.10, *p* < 0.001); social anxiety predicted academic burnout positively (*β* = 0.42, *t* = 10.23, *p* < 0.001) and self-control negatively (*β* = −0.36, *t* = −9.61, *p* < 0.001); self-control predicted academic burnout negatively (*β* = −0.46, *t* = −10.86, *p* < 0.001) (see Figure 2 for the standardized path model).

Furthermore, a bias-corrected Bootstrap test for mediating effects and confidence interval estimation was used with 1,000 times repeated samples. Finally, the findings demonstrated that the separate mediation effect of social anxiety between parental phubbing and adolescents’ academic burnout was significant (*β* = 0.098, SE = 0.022, 95% CI [0.060, 0.145]). Parental phubbing and adolescents’ academic burnout were separately mediated by self-control, and this impact was significant (*β* = 0.065, SE = 0.023, 95% CI [0.025, 0.116]). In the meantime, the serial mediation effect of social anxiety and self-control was also significant (*β* = 0.039, SE = 0.012, 95% CI [0.020, 0.068]). Specific indirect effects and 95% CI are shown in Table 2.

**Table 2:** Bootstrap analysis of the direct and indirect effects of each path.

Paths	Estimate	Relative effect	95% confidence intervals	
			Lower	Upper
Direct effect	0.097	32.6%	0.019	0.183
Parental phubbing – social anxiety – academic burnout	0.098	32.9%	0.060	0.145
Parental phubbing – self-control – academic burnout	0.065	21.8%	0.025	0.116
Parental phubbing – social anxiety – self-control – academic burnout	0.039	13.1%	0.020	0.068

Bootstrap 95% CI does not include 0, representing that the path is statistically significant.

## 4. Discussion

In the current research, we found that parental phubbing, social anxiety, self-control, and adolescents’ academic burnout were significantly correlated in pairs. These indicated that parental phubbing is associated with high social anxiety and low self-control in adolescents, all of which increased their risk of experiencing academic burnout. Based on the Limited Resource of Self-control theory, we further identified the internal mechanism of parental phubbing impacting academic burnout through the chain mediating roles of social anxiety and self-control in adolescents.

### 4.1. Parental phubbing on adolescents’ academic burnout

He et al. (2022) found that the higher the phubbing exhibited by parents, the higher the level of academic burnout experienced by adolescents. The present research supported He et al.’s findings that parental phubbing will lead to a decreased interest in learning, reduced sense of achievement, and burnout problems in adolescents.

Family environment is an important factor influencing adolescents’ academic burnout (Luo et al., 2020). Parents’ behavior of playing with their cell phones in front of adolescents may make them feel rejected and neglected by their parents (Roberts and David, 2016; David and Roberts, 2017). When individuals experience rejection by parents, they will feel anxious, angry, insecure, and unloved, resulting in distorted representations of the world around them, leading to a range of problem behaviors (Rohner et al., 2005). Earlier researchers have also reported children who are rejected and neglected by their parents are more prone to experience academic burnout (Luo et al., 2016). In addition, parents who regularly play with their cell phones at home while neglecting work and study will set their children a bad example, which will lead them to perform the same phubbing actions (Niu et al., 2020), and make them have a wrong cognition and attitude towards learning.

### 4.2. The mediation role of social anxiety and self-control

This research discovered that social anxiety mediated the relationship between parental phubbing and adolescents’ academic burnout. In other words, parental phubbing predicted adolescents’ academic burnout by raising social anxiety. Previous findings have shown that parental phubbing leads to adolescent social anxiety (Zhang et al., 2022). This is mainly due to the fact that parental phubbing leads to a decrease in parent–child communication and parent–child relationships (Chotpitayasunondh and Douglas, 2016, 2018; Kildare and Middlemiss, 2017). Failing to interact with parents, who are children’s initial interpersonal contacts, would cause children to lose confidence in social activities, which in turn leads to social anxiety (Merikangas et al., 2003). Philosophically speaking, human beings are social animals and individuals cannot survive alone without society, and learning activities also need to take place in a social environment. Adolescents with social anxiety are afraid of making friends with others, and their poor social skills prevent them from forming good peer relationships with others, which further contributes to academic burnout (Zhou et al., 2022).

In the current study, we found that parental phubbing would reduce adolescents’ capacity for self-control, which results in increased degrees of academic burnout. Previous studies have discovered that self-control and academic burnout were negatively associated (Love et al., 2020), with those with higher self-control having lower academic burnout (Seibert et al., 2016). So self-control may play a key role in adolescents’ learning. The act of learning requires plenty of self-control resources (Tangney et al., 2004), while these resources will be consumed by external environmental factors and internal negative emotional states (Beaver et al., 2009; Blackhart et al., 2015). According to the Limited Resources of Self-control theory, the resources of self-control are not inexhaustible (Muraven and Baumeister, 2000; Baumeister, 2003). So when the resources were consumed by environmental factors, the remaining resources are insufficient to cope with the needs of learning, and then will lead to learning problems. Parenting and family environments have long been perceived as significant to the development of self-control (Botchkovar et al., 2015; Davis et al., 2017; Li et al., 2019; Luo et al., 2020). As a negative parenting style, parental phubbing was a significant sign of rejection and ignoring (Roberts and David, 2016; David and Roberts, 2017), which can deplete huge control resources and then result in self-control failure (Davis et al., 2017).

Additionally, this research discovered that social anxiety and self-control act as a chain mediator between parental phubbing and adolescents’ academic burnout. The result of this study further supports the findings that social anxiety leads to reduced self-control (Kashdan et al., 2011; Blackhart et al., 2015). Parental phubbing can make adolescents have negative expectations about interpersonal interactions and then trigger social anxiety (Zhang et al., 2022). According to the Cognitive Model of Social Anxiety (Hofmann, 2007), those with social anxiety constantly assess the “threat” in the current social situation, which may be a real or imagined potential audience. But whether there is an audience or not, when confronted with a “threat,” the individual will turn his or her attention inward and focus heavily on their self, which consumes plenty of self-control resources. In addition, social anxiety, as a negative emotion, also constantly consumes self-control resources, leading to reduced self-control. Learning activities also require control resources, and insufficient resources result in control failure, which raises the risk of academic burnout (Seibert et al., 2016).

### 4.3. Insights and limitations

There has been a lot of scholarly attention to the effects of this new parenting style on child and adolescent development, even though the term “parental phubbing” has just recently emerged with the fast expansion of internet mobile devices (Kildare and Middlemiss, 2017; Mcdaniel and Radesky, 2018a). To date, however, only a few studies focused on the effects of parental phubbing on adolescents’ academic burnout (He et al., 2022). Consequently, based on the Limited Resources of Self-control theory, the current study incorporates interpersonal variables (social anxiety) and personal factors (self-control) into the model to reveal more comprehensively the mechanism of parental phubbing affecting adolescents’ academic burnout as well as broaden the research in this area.

In addition, there are still several limitations that need to be noted. Firstly, as a cross-sectional study, although the current research found a correlation between parental phubbing and adolescents’ academic burnout, there was not enough evidence to determine the development of this relationship over time and the causal association. It is necessary for future research to take a longitudinal study into account to thoroughly examine the causal link and dynamic changes. Secondly, we focused on the effect of parental phubbing on adolescents’ academic burnout but did not research the influences of paternal and maternal phubbing separately. Fathers and mothers often play different roles in the family, and their phubbing actions may have different effects on adolescents. Finally, only the questionnaire method was used in this study for measurement, which is subjectively influenced by the participants, and students’ attitudes to devices may also have some influence on the results. In the meantime, the social approval effect may obscure the true situation of the subjects, and future studies can use more objective methods to collect data.

## 5. Conclusion

This research found that: (1) parental phubbing and adolescents’ academic burnout were significantly and positively associated. (2) Parental phubbing can influence adolescents’ academic burnout by the separate mediation effects of social anxiety and self-control. Social anxiety was positively associated with adolescents’ academic burnout, whereas self-control can negatively predict adolescents’ academic burnout. (3) Parental phubbing predicts adolescents’ academic burnout through the chain mediation effect of social anxiety and self-control. Social anxiety affects self-control negatively. These results provide some practical inspiration for parents and teachers to understand the reasons for adolescents’ declining interest in learning and lack of motivation, and how to intervene in their academic burnout. Firstly, parents need to take the problem of declining academic performance and academic burnout among adolescents seriously enough, but instead of just blaming the students, they should understand that adolescents’ development is influenced by their parents and look more for the causes of the problem from themselves. Secondly, parents should pay attention to reducing the use of internet devices to prevent addiction and consciously avoid using cell phones in front of their children. Even if they cannot avoid using cell phones in the family due to work, they should let their children know the real reason and set a good example for them to work actively and study hard. Thirdly, parents should pay attention to more in-depth communication with their children, encourage them to communicate with others, and develop good social skills. At the same time, schools should also pay attention to shaping a learning atmosphere conducive to interpersonal communication and promoting good peer relationships among students. Fourthly, parents must emphasize the critical role that self-control plays in their adolescents’ learning difficulties. They need to concentrate on improving their kids’ capacity for self-control to strengthen their risk tolerance.

## Data availability statement

The raw data supporting the conclusions of this article will be made available by the authors, without undue reservation.

## Ethics statement

The studies involving human participants were reviewed and approved by The Ethics Committee of Anhui Normal University. Written informed consent to participate in this study was provided by the participants’ legal guardian/next of kin.

## Author contributions

YJ and RH contribute to the title selection and design of this research. RH undertook the guidance and revision of the article. YJ wrote the first draft of the article. LL and YJ constructed the database, performed statistical analysis, and translated the article. All authors contributed to the article and approved the submitted version.

## Conflict of interest

The authors declare that the research was conducted in the absence of any commercial or financial relationships that could be construed as a potential conflict of interest.

## Publisher’s note

All claims expressed in this article are solely those of the authors and do not necessarily represent those of their affiliated organizations, or those of the publisher, the editors and the reviewers. Any product that may be evaluated in this article, or claim that may be made by its manufacturer, is not guaranteed or endorsed by the publisher.
